# Nurses in the triage of the emergency department: self-compassion and empathy*

**DOI:** 10.1590/1518-8345.3049.3151

**Published:** 2019-07-18

**Authors:** Roberta Maria Savieto, Stewart Mercer, Carolina Carvalho Pereira Matos, Eliseth Ribeiro Leão

**Affiliations:** 1 Faculdade Israelita de Ciências da Saúde Albert Einstein, São Paulo, SP, Brasil.; 2 University of Glasgow, Institute for Health and Wellbeing, Glasgow, Escócia.; 3 Universidade de São Paulo, Escola Politécnica, São Paulo, SP, Brasil.; 4 Hospital Israelita Albert Einstein, Research Institute, São Paulo, SP, Brasil.

**Keywords:** Empathy, Emergency Service Hospital, Triage, Nursing, Emergency Nursing, Psychometrics, Empatia, Serviço Hospitalar de Emergência, Triagem, Enfermagem, Enfermagem em Emergência, Psicometria, Empatía, Servicio de Urgencia en Hospital, Triaje, Enfermería, Enfermería de Urgencia, Psicometría

## Abstract

**Objective:**

to adapt the Consultation and Relational Empathy Measure (Brazilian version) for nurses; to evaluate the concurrence between empathy self-reported by nurses and that perceived by patients; To correlate self-compassion to the empathy self-reported by nurses and perceived by patients.

**Method:**

seven specialists validated the Consultation and Relational Empathy Measure Nurses (Brazilian version)' adaptation by original author's authorization. A sample with 15 triage nurses and 93 patients they admitted to the Emergency Department of a philanthropic private hospital were interviewed according to the following instruments: Consultation and Relational Empathy Measure – Nurses (Brazilian version) and the Self-Compassion Scale (Brazilian version).

**Results:**

the psychometrics properties of Consultation and Relational Empathy Measure – Nurses (Brazilian version) showed appropriate internal consistency (*Cronbach's alpha=0,799).* The evaluation of empathy provided by the patients was better than that self-reported by the nurses *(p<0,001)*. The nurses with higher level of self-compassion also showed higher empathy scores *(p=0,002)*.

**Conclusion:**

our results confirmed the psychometrics properties' adequacy of Consultation and Relational Empathy Measure – Nurses (Brazilian version), allowing to compare empathy scores embased at same parameters. Self-compassion showed to influence self-reported empathy.

## Abstract (pt)

**Objetivo:**

este estudo adaptou e validou a escala *Consultation and Relational Empathy Measure* (versão brasileira) para avaliação da empatia por enfermeiros; avaliou a concordância entre empatia autorreferida pelos enfermeiros e a percebida pelos pacientes e correlacionou autocompaixão com empatia autorreferida dos enfermeiros e percebida pelos pacientes.

**Método:**

sete juízes validaram a adaptação da escala citada para a *Consultation and Relational Empathy Measure Nurses* (versão brasileira) com anuência do autor da escala original. Uma amostra de quinze enfermeiros e 93 pacientes do Serviço de Emergência de um hospital privado filantrópico, foram avaliados utilizando a *Consultation and Relational Empathy Measure Nurses* (versão brasileira) e a Escala de Autocompaixão (versão brasileira).

**Resultados:**

as propriedades psicométricas da *Consultation and Relational Empathy Measure Nurses* (versão brasileira) demonstraram consistência interna adequada *(alfa de Cronbach de 0,799).* A empatia na visão dos pacientes foi mais bem avaliada do que a autorreferida pelos enfermeiros *(p<0,001)*. Enfermeiros mais autocompassivos apresentaram maiores escores de empatia *(p=0,002)*.

**Conclusão:**

os resultados deste estudo confirmaram a adequação das propriedades psicométricas da *Consultation and Relational Empathy Measure Nurses* (versão brasileira), o que permite a comparação da empatia com a escala para pacientes a partir dos mesmos parâmetros de avaliação. A autocompaixão influenciou a empatia autorreferida pelos enfermeiros.

## Abstract (es)

**Objetivo:**

este estudio adaptó y validó la versión brasileña de la escala para enfermeros: *Consultation and Relational Empathy Measure*; evaluó la conformidad entre la empatía auto declarada por las enfermeras y la percibida por los pacientes; correlacionó la autocompasión con la empatía auto declarada por los enfermeros y la percibida por los pacientes.

**Método:**

siete jueces validaron la adaptación de la escala citada para la *Consultation and Relational Empathy Measure Nurses* (versión brasileña) con anuencia del autor de la escala original. Una muestra de 15 enfermeros y 93 pacientes ingresados en el Departamento de Emergencias de un hospital privado filantrópico fueran evaluados utilizando la *Consultation and Relational Empathy Measure Nurses* (versión brasileña) y la Escala de Autocompasión (versión brasileña).

**Resultados:**

las propriedades psicométricas de la *Consultation and Relational Empathy Measure Nurses* (versión brasileña) demonstraron consistencia interna adecuada *(alfa de Cronbach = 0,799).* La empatía en la visión de los pacientes fue mejor evaluada que la autorreferida por los enfermeros *(p<0,001)*. Enfermeros más autocompasivos presentaron mayores escores de empatía *(p=0,002)*.

**Conclusión:**

los resultados de este estudio confirmaron la adecuación de las propriedades psicométricas de la *Consultation and Relational Empathy Measure Nurses* (versión brasileña), lo que permite la comparación de la empatía con la escala para pacientes a partir de los mismos parámetros de evaluación. La autocompasión influenció la empatía autorreferida por los enfermeros.

## Introduction

The experience of health service patients regarding the care they receive from the health professionals is highly relevant for the evaluation and development of those services^(^ ^1^ ^)^. Attention to their reports and impressions is essential for the creation of a health system focused on the patient with an emphasis on quality and strengthened by studies and public policies^(^ ^2^ ^)^. Thus, the meaning of care and the perception of qualified assistance to patients in the emergency department is influenced by the quality of interpersonal relations, empathy, the professionals being open to talk and listen, and the validation of the information they provide^(^ ^3^ ^–^ ^4^ ^)^.

Additionally, we can observe a discrepancy between what emergency nurses consider important, that is the absolut readiness in the treatment of physiological disorders and what patients and their family members realize as fundamental, like communication abilities, critical thinking and sensitivity^(^ ^5^ ^)^. In this sense, the literature has highlighted the essentiality of the nurse's work in the emergency service, as it provides quality in health care, which makes them different from other professionals because they have the technical ability combined with the interpersonal skills^(^ ^5^ ^–^ ^6^ ^)^. Consequently, nurses should have communication skills to provide the best assistance possible, as they are the core of the units and must relate to individuals that play several roles in the workplace, as well as to the patients themselves.

To meet these demands and reinforce the nursing attributions, empathy emerges as a strategy, since it provides the sensation of task accomplishment for the professional and greater satisfaction for the patients and their family members^(^ ^7^ ^–^ ^11^ ^)^. Even though the concept of empathy encompasses several aspects, the individual's capacity to understand the feelings of another person and show the other this understanding represents its core^(^ ^7^ ^–^ ^8^ ^)^. It is embased on three pillars: cognitive (the intellectual ability to understand feelings); affective or emotional (the ability to put oneself in another person's shoes, as in the English expression *“walk a mile in his moccasins”*); behavioral (represented by effectively communicating the understanding of the situation)^(^ ^12^ ^)^.

Empathy and compassion are complementary characteristics fundamental for the process of care in Nursing. While empathy promotes understanding the situation of the other, compassion favors acting to relieve the suffering the situation brings about^(^ ^13^ ^–^ ^14^ ^)^. Self-compassion is strongly related to compassion for others. Hence, with higher self-compassion, the professional can both be connected with the needs of the other and protect themselves from the emotional burnout caused by this empathic connection^(^ ^14^ ^)^.

The discussion of the self-compassion concept is relatively recent in the West. It began to appear in the literature less than two decades ago and is in line with Buddhist principles. According to this reference, self-compassion comprises three main components: the balance between kindness to oneself and self-criticism, which is related to our capacity of being more gentle with ourselves without passing painful self-judgments, and being kinder about our attitudes; sense of humanity and isolation, regards the fact that we recognize ourselves as humans, therefore liable to mistakes, so as to put ourselves in the same position of any other person, without isolating ourselves with our mistakes and; the mindfulness-fixation relationship, which means the person being aware of and focused on the present moment, nor ignoring neither constantly revising life problems^(^ ^15^ ^)^.

Studies on the empathy in emergency services are scanty, as are the instruments that evaluate such parameter in the dyad nurse-patient. Until this study, there is no specific scale for the self-evaluation of empathy from health professionals, neither from nurses, considering the same evaluation parameters. Some instruments evaluate empathy from the perspective of patients^(^ ^11^ ^)^, others from the perspective of physicians^(^ ^16^ ^)^, health professionals^(^ ^17^ ^–^ ^18^ ^)^ and students^(^ ^17^ ^)^. Instruments that allow both evaluations are rare, and few scales are available for the use in Brazil^(^ ^11^ ^,^ ^19^ ^)^.

The Consultation and Relational Empathy (CARE) Measure was initially devised to allow for the evaluation patients made of the empathy of the physicians who attended them^(^ ^20^ ^)^, being later extended to other health professionals. It was properly translated and adapted to the Brazilian population, proving easy to be understood by the patients, and thus indicated to evaluate empathy in the context of health service^(^ ^11^ ^)^. The Self-Compassion Scale (SCS) was created and validated in the USA in 2003 to evaluate self-compassion^(^ ^21^ ^)^. This instrument was largely advertised and used all over the world, being translated and validated in many countries, including Brazil^(^ ^22^ ^)^.

For this reason, the objectives of this study were: to adapt the CARE Measure (Brazilian version) that is applied for patients to evaluate the professionals' empathy, for CARE Measure – Nurses (Brazilian version), that turns possible the empathy' self-evaluation by nurses; to evaluate the concurrence between the empathy self-reported by the nurses and that perceived by the patients in the emergency department assistance and to correlate the self-compassion to the empathy reported by the nurses and perceived by the patients.

## Method

This study was developed in a Master's Nursing Professional Program, carried out in two phases: 1) the adaptation of the CARE Measure (Brazilian version) for nurses and 2) the validation of the CARE Measure – Nurses (Brazilian version). The data were collected in an emergency unit of a philanthropic private hospital with over 500 beds in the city of São Paulo, Brazil, between October and November 2015 and met all the ethical criteria established by the institution and the Brazilian legislation (number CAAE 39441114.2.0000.0071).

In this service the triage nurse classifies the patients based on the Emergency Severity Index (ESI), according to the severity and the number of resources (exams, medication) required for their treatment, in addition to the medical specialty. The index ranges from 1 to 5, where 1 is the most severe, requiring immediate attention (such as cardiorespiratory arrest); 2 poses great risk and is inserted in the institutional protocols (cerebral vascular accident, acute myocardial infarction and sepsis); 3 requires two or more resources for the investigation of the condition; 4 is more easily treated, requiring a simple solution and one single resource; and 5, when patients only receive medical evaluation and are discharged straight from the physician's office^(^ ^23^ ^)^.

In Stage I, Stewart Mercer, author of the CARE Measure, authorized us to use it and to make alterations for emerge CARE Measure – Nurses (Brazilian version). We too have asked, and achieved, permission for change the CARE Measure (Brazilian version) that was translated and adapted by José Antonio Baddini Martinez. The new instrument named CARE Measure – Nurses (Brazilian version) was evaluated by a committee of seven experts specialized in communication and emergency so we could complete the content validation^(^ ^24^ ^–^ ^25^ ^)^. They used an online questionnaire available at the *Survey Monkey*®, a platform where they should agree or disagree with the alteration proposed, justify their choice and make a suggestion.

Two sessions of analyses were necessary before the experts reached an agreement of 80%, as required in the literature on this kind of work^(^ ^25^ ^–^ ^26^ ^)^, after which the CARE Measure – Nurses (Brazilian version) was available for use in the second stage of the study. To assess the internal consistency and the reliability of the instrument we used the Cronbach's alpha test that is capable to detect if the scale can evaluate what is proposed to measure under any circumstances^(^ ^27^ ^)^.

Data collection was carried out in Phase II with the following target populations: nurses who had been working in the triage sector for at least one year, except those who worked in pediatrics or were on a leave; patients attended by those professionals, 18 to 65 years old, classified as levels ESI 3, 4 and 5, with cardiovascular, respiratory, gastrointestinal, gynecological conditions. They were either private patients or had a health plan. The exclusion criteria were: patients classified in the triage as ESI 3, 4 or 5 who evolved to 1 or 2; those with neurological conditions (except migraine) due to possible mental and cognitive alterations; individuals with communication deficit or any other disorders that made it impossible for them to answer the questionnaire, and foreigners.

The nurses answered a sociodemographic questionnaire; the CARE Measure – Nurses (Brazilian version), and the Self-compassion Scale (Brazilian version). The patients answered a sociodemographic questionnaire and the CARE Measure (Brazilian version).

The sample, made by convenience, comprised 15 nurses and 93 patients. In order to evaluate concurrence between the nurses' self-reported empathy and that perceived by the patients they attended we considered nine nurses and 67 patients, as we established a minimum number of four patients per nurse, which was the minimum number of patients evaluated by each nurse capable of being adequate to the linear mixed model used in the statistical analysis of these data so we could take into account the dependence between the evaluations different patients made of the same nurse. Moreover, according to the recommendations of the authors of the original and translated CARE Measure^(^ ^11^ ^,^ ^20^ ^)^ we excluded patients who chose “does not apply” for more than two statements.

To analyze the data, we used the programs Statistical Package for the Social Sciences (SPSS) and Stata, with level of significance at 5%. The categorical variables were described by absolute frequencies and percentages, while the numerical ones by summary-measures as mean and standard deviation (SD), median and interquartile interval (IQI), in addition to minimum and maximum values. The correlation between the self-compassion scores of the nurses and the empathy perceived by the patients was evaluated by the weighted correlation coefficient while the correlation between the nurses' self-reported self-compassion and empathy was evaluated by the Pearson's correlation coefficient.

## Results

**Figure 1:**
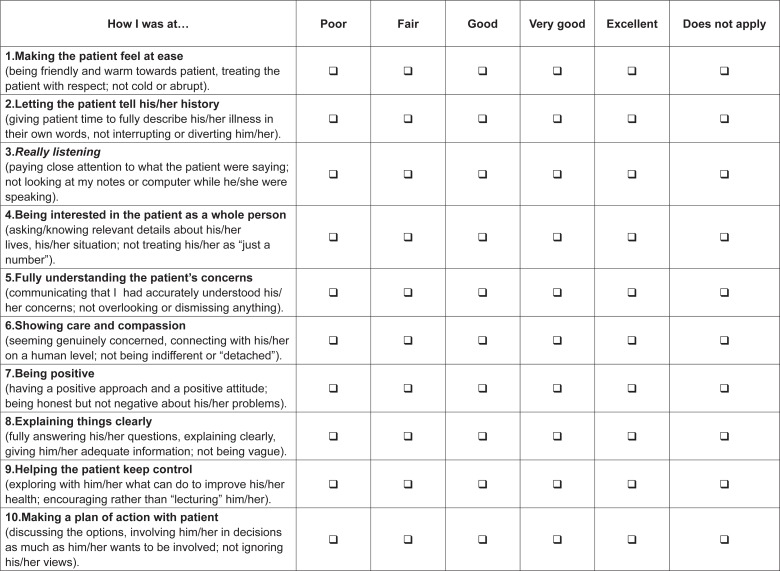
Consultation and Relational Empathy (CARE) Measure – Nurses (Brazilian version), 2016

In Stage I, we adapted the refereed scale and the CARE Measure – Nurses (Brazilian version) emerged (Figure 1). The instrument showed psychometrics properties 'adequacy adequacy with *Cronbach's alpha yielded 0,799* (> 0,70) which indicates high internal consistency^(^ ^28^ ^)^.

In Stage II, we obtained the sample of 15 nurses was composed by 86,7% of females, ages between 25 and 43 years, mean age of 33,4 years *(SD=5,2 years).* The time they had spent on their professional formation ranged from three to 10 years *(SD=4,8 years)*, and most of them graduated from private institutions (80,0%). All of them reported having taken at least one post-graduation course. They had been working at the emergency department of the institution between one and 13 years with a median of five years *(IQI: 4 to11 years)*.

The sample of patients was composed by 58,1% of females, ages ranging from 18 to 64 years, with a mean of 40,6 years *(SD=10,2 years)*. Regarding schooling, 88,2% of the patients had at least a college degree. They were attended predominantly in the morning (48,4%) and afternoon (47,3%), and 72% of the patients had 3 in the ESI at triage.

In the CARE Measure, the values of the individual items are added, resulting in final scores between 10 and 50^(^ ^11^ ^)^. In the sample of nurses, the scores ranged from 25 to 45, with a mean of 37,9 *(SD=5,2)*, while the score of empathy perceived by the patients varied from 18,8 to 50,0, with a mean of 42,4 *(SD=8,3)*.

**Figure 2:**
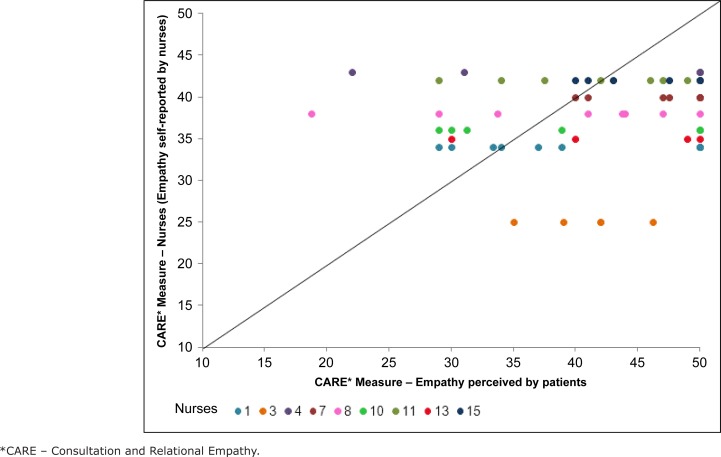
Consultation and Relational Empathy (CARE*) Measure scores of empathy self-reported by the nurses and that perceived by the patients. São Paulo, SP, Brazil, 2015

Figure 2 shows the dispersion between the scores of empathy self-reported by the nine nurses and that perceived by the 67 patients they attended. Each shade of gray represents one participating nurse, and a diagonal line represents cases in which the scores of the nurse and that of the patient were identical.

The difference between the empathy self-reported by the nurses and that perceived by the patients was significant *(p<0,001)*. What means that the difference between the self-perception of nurses' empathy and that the patient they attend did not happen by chance.

The estimated *mean* of this difference is *4,78*, with a confidence interval between *2,58* and *6,97*, which shows that the patients evaluated the nurses as more empathic than those professionals evaluated themselves.

The analysis of the response of the nurses to each statement of the Self-compassion Scale (Brazilian version) shows a good result – *mean of 3,51 (SD=0,48)* between 0 and 5. The evaluation of each dimension of the instrument, however, showed little compassionate responses.

The degree of correlation was assessed by the weighted correlation coefficient, corrected by the repetitions among nurses. We did not find evidence of correlation between the nurses' scores of self-compassion and the empathy perceived by the patients *(r=0,38; p=0,309)*.

**Figure 3:**
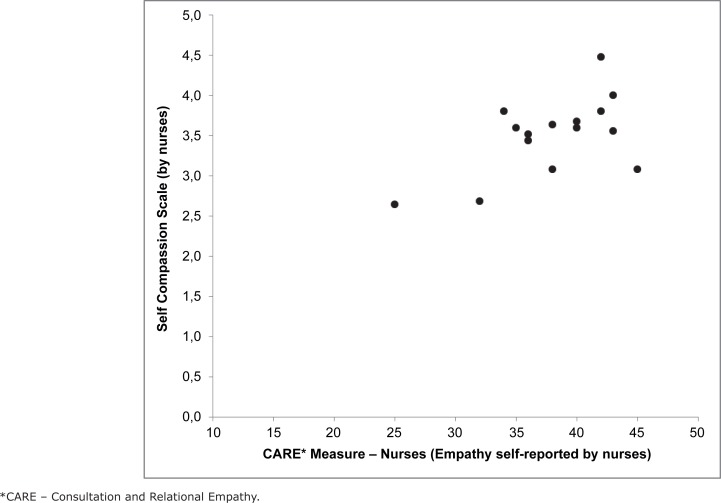
Scores of Self-Compassion Scale (Brazilian version) and Consultation and Relational Empathy (CARE*) Measure Nurses by nurses. São Paulo, SP, Brazil, 2015

The correlation between self-compassion and empathy reported by the nurses was considered for the total sample of 15 professionals, as presented in Figure 3.

We observe a trend according to which high scores of empathy self-reported by the nurses were associated with higher scores of self-compassion. The degree of correlation was evaluated by the Pearson's correlation coefficient, having showed a strong correlation between the scores of self-compassion and those of empathy because the closer to one the greater the strength of the correlation between the variables.*(r=0,72; p=0,002)*.

## Discussion

In Stage 1 we adopted a pioneer measure as we did not find any studies whose focus of discussion was the nurses and their patients and adapted an adequate scale. So, we configure the importance of the present study, to allow the comparison between the empathy of nurses and patients based on the same instrument. The authors of the validation of the CARE Measure in Brazil^(^ ^11^ ^)^ did not find correlations between the results of the CARE Measure and two other scales of empathy self-evaluation, the Interpersonal Reactivity Index (IRI) and the Empathy Inventory (EI). Therefore, we found significant encouragement to develop a scale that would be able to evaluate and compare, using the same parameters, the empathy self-reported by the nurses and that perceived by the patients, remembering that this process had not been carried out specifically with nurses.

Our findings reached statistically significant differences between self-reported empathy and that perceived by the patients, so that the nurses considered themselves less empathic than their patients did. We found similar conclusions in a study, that among other data, observed that the medical specialty denominated emergency is considered the one that might entail the highest level of emotional exhaustion, due to the severe condition the patients present and the high demand from the professionals, which results in a lower level of empathy and higher compassion fatigue of those professionals^(^ ^29^ ^)^. Thus the data suggest that the self-evaluation seems to be attached to the nature of the specialty rather than to the exactly professional category and configures a theme that deserves to be more explained in future researches.

Additionally, we know that the professionals working in the emergency unit prefer to attend patients in severe condition instead of the less critical ones, who could even be treated at the outpatient unit^(^ ^5^ ^)^, as in the case of the professionals evaluated in this study. The lower score of self-reported empathy, hence, might be associated with the profile of the patients, who present less severe complaints that do not conform to the profile expected from the professionals trained in the emergency unit studied.

On the other hand, the teaching of empathic skills also requires a deeper discussion, since it is already known that the availability of knowledge does not necessarily make an individual change their behavior. In the area of health, it is also possible to make an analogy with the practice of hands hygiene which, in spite of deemed the best method to fight infections, is the target of campaigns and frequent training due to inadequate techniques or its non-execution^(^ ^30^ ^)^. Currently there are courses and training all over the world that claim to offer syllabus to anyone (in the health area or not) that will make the individual more empathetic and compassionate, and able to change their relationships^(^ ^31^ ^–^ ^33^ ^)^.

Systematic review on the efficacy of empathy trainings showed, among other results, that the longest post-intervention evaluation lasted six months and that the performance of objective measures (as scores) of empathy showed a better result than the self-report, reinforcing the importance of development and implementation of objective instruments to evaluate subjective skills and the need that the training content always include the cognitive, affective and behavioral pillars^(^ ^34^ ^)^, which seems to be closest to the reality of the professionals who participated in this study and the results we found.

Some researchers aim at demonstrating that it is possible to teach empathy and compassion to any human being, independently of the age group, due to the constant condition of neural plasticity, provided that there is continuous socioemotional stimulation. So based on genetic factors, brain maturation and previous relationship experiences, it is possible to modulate the skills of empathic perception, depending on the intensity, continuity and frequency of the challenges and interpersonal simulations^(^ ^35^ ^)^. For the compassionate behavior of medical students as well as that of other health professionals to be improved, they should be given ample opportunity to have critical self-evaluation. Moreover, their professors should be role models of teaching and assistance^(^ ^36^ ^)^.

However, there is still controversy as to how this mechanism works. Even considered as a personality trait alone, empathy, in the context of health service, is permeated by factors alien to the individual, such as social and organizational resources^(^ ^37^ ^)^. Other factors yet may influence the empathic relation between individuals. By means of the evaluation of brain waves, researchers observed that our empathic behavior depends on external factors like ethnic groups^(^ ^38^ ^)^. They also found out that those who have the highest level of empathy can perceive a larger variety of facial expressions and, consequently, sense the emotions of the others^(^ ^39^ ^)^.

Hence, there are those who defend that a possible approach to improve empathic behavior would be investing on training for the perception of facial expressions, as the basic emotions (fear, surprise, anger, disgust, sadness, despise and happiness) constitute universal face movements and cannot be faked^(^ ^40^ ^)^. Nevertheless, perceiving the emotion of others by their facial expression alone does not guarantee a compassionate and empathic behavior, as this would be related to the cognitive pillar of empathy, which means understanding the situation of the other, and contemplating the emotional and behavioral aspects would still be required.

The nurse is the professional pointed by the patients of the emergency/urgency service as the one who can provide updated information, listen to their concerns and improve the interpersonal relations of those involved in the care^(^ ^41^ ^)^. The patients also value the sensitivity of the nurses in the emergency unit^(^ ^5^ ^)^. In this respect, the results of the present study seem to be contrary to those proposed in the literature, as several patients implied, in their responses to the CARE Measure, that they did not recognize the need of continuous follow-up from the nurse. Some possibilities may account for that discrepancy: higher psychosocial autonomy from the patients, who already have favorable socioeconomic conditions, or still their undervaluation of the nurse, as those patients consider the physician the only one responsible for the care planning and follow-up, even in a multi-professional environment^(^ ^42^ ^)^.

The creators of the CARE Measure reinforce the importance of the scale on the influence the professional exerts on the treatment proposed to the patients. Its items are based on the understanding that empathy, in the clinical context, involves the ability to understand the sensations of the patients (cognitive aspect), put themselves in their place (affective/emotional aspect), make the patients aware of this understanding and act in a therapeutic way to help them (behavioral aspect)^(^ ^20^ ^)^. Consequently, the three first items of the CARE Measure - Nurses (Brazilian version) are not only similar to the necessary attributes that make a cordial social coexistence, but they represent fundamental factors for the development of the empathic process as well.

The analysis of the responses of the nurses to the Self-Compassion Scale (Brazilian version) demonstrated that in the first set of dimensions (sense of humanity x isolation) the difficulties faced and the mistakes made by those professionals might generate feelings of frustration and, consequently, solitude and isolation. In the second set of dimensions of the scale (kindness to myself x self-criticism), we notice the professionals are able to be kind with themselves, but they exhibit an important trait of self-criticism. In the last combination of dimensions (mindfulness X fixation), the responses showed that keeping focus on the problems is a controversial issue among those professionals, making one to wonder how much they actually allow themselves to “feel down”.

Hence, this self-demanding characteristic of the nurses stands out and may also be related to the isolation in the previous set of dimensions, as those who demand too much from themselves and criticize themselves too much might feel isolated when something does not happen as expected^(^ ^43^ ^)^. Therefore we noticed some incongruences in our findings because despite the self-compassion evaluation' results above average, when we evaluate the subdimensions disconnectedly, we observed answers less self-compassionate.

For that reason, one should be cautious when directly associating the result of the scale with the levels of self-compassion. We found study that also recommends that the differences between positive and negative statements be better explored, as the indication of results by the subdimensions^(^ ^44^ ^)^. Based on this recommendation, and despite the results we found, we should question whether the nurses are really self-compassionate. Considering this is an issue of recent interest to health professionals, and the multidimensionality it involves, there is still a long way to go before the accuracy in the evaluation of compassion within health organizations is reached.

The literature also points that nurses at urgency/emergency units tend to feel much pressure and the obligation of not failing, which results in a strong self-demanding behavior^(^ ^4^ ^,^ ^45^ ^)^. Therefore, we might understand those response as part of the work process and a reflection of the unit they are inserted in. The concern of managers, educators and professionals about the self-care of the nurses and the maintenance of their self-compassion has been recently growing, as these would reflect on their showing compassion to their patients.

It is adamant that professionals be aware that taking care of themselves and being self-compassionate is not selfishness^(^ ^14^ ^,^ ^46^ ^)^, and that there are strategies to help them reach this awareness, such as meditation and maintenance of mindfulness^(^ ^47^ ^–^ ^48^ ^)^. Hence, it seems that nurses need to be convinced that they, too, deserve to be taken care of, which corroborates our findings, as the trend to isolation and strict self-criticism appear even in “apparently good self-evaluations” of compassion and empathy.

This realization might justify the lack of relation found in this study between the level of self-compassion of the nurses and that perceived by their patients. We may question whether for self-compassion to be related to the empathy perceived by the patients, the mean value of 3,51 should be higher, as we could observe important issues associated with the absence of self-compassion, or even whether this relation was not established due to the real low self-compassion of the nurses, masked by the final score above the mean. The contradictions previously discussed lead to this assumption and should be further investigated.

Compassion and empathy are related, in the sense that even the lowest level of self-compassion develops an empathic behavior and generates compassionate attitudes towards others. Hence, the discomfort caused by the suffering of others may bring about empathy from the nurse, and the relief of that suffering give satisfaction and personal and professional fulfillment. Therefore, those who are more satisfied are more self-compassionate. In this way a virtuous cycle of self-compassion-empathy-compassion takes place^(^ ^29^ ^)^.

## Strengths and Limitations

The major contribution of this study is undoubtedly the availability of the CARE Measure – Nurses (Brazilian version) for the self-evaluation of nurses and, similar what have been done with the CARE Measure, the new instrument fits to self-evaluation of other professionals in health care too. In this way, from now, it is possible to verify and check the nurses (or other health professionals) self-empathy and that perceived by patients with the same instrument, with the same theoretical reference.

However, it was not possible to perform construct validity by the factorial analysis since, according to the reference we used^(^ ^49^ ^)^, the sample size suggested is that the number of observations should be at least five fold the number of variables, and that this analysis should not be used with samples lower than 50 observations. Therefore, as our study was carried out with 15 nurses (with no possibility of enlarging the sample due to the number of professionals available and the time frame for the performance of the study), we could not meet this requirement.

The application of the CARE Measure – Nurses (Brazilian version) was made with 15 professionals working exclusively in triage, not professionals from other sectors of the emergency unit. Consequently, the results may not represent the whole unit, which is why further studies are required that include nurses from other sectors, as well as studies with patients and professionals of other types of emergency units, as in public services, to broaden the reach of the results and the discussion of this issue. For this reason we strongly suggest that CARE Measure – Nurses (Brazilian version) be applied in another care units and with other professionals, just like CARE Measure.

## Conclusion

The adaptation of the CARE Measure – Nurses (Brazilian version) was devised for nurses in the triage of an Emergency Unit and showed psychometrics properties 'adequacy of content validity and high reliability.

We detected a statistically significant difference between the empathy self-reported by the nurses and that observed by the patients, with the patients making a better evaluation, in other words, the patients perceived the nurses more empathic than themselves self-evaluation. There was no correlation between the self-compassion of the nurses and the empathy perceived by the patients, but rather evidence of the correlation between compassion and empathy self-reported by the professionals.
